# Supplementary material for: Transcriptome Analyses Identify Deregulated MYC in Early Onset Colorectal Cancer
Source: Biomolecules. 2022 Sep 2;12(9):1223. doi: 10.3390/biom12091223 (PMC9496520; doi:10.3390/biom12091223)
Supplement: Supplementary file 1 [file biomolecules-12-01223-s001.zip › biomolecules-1814831-supplementary.pdf]

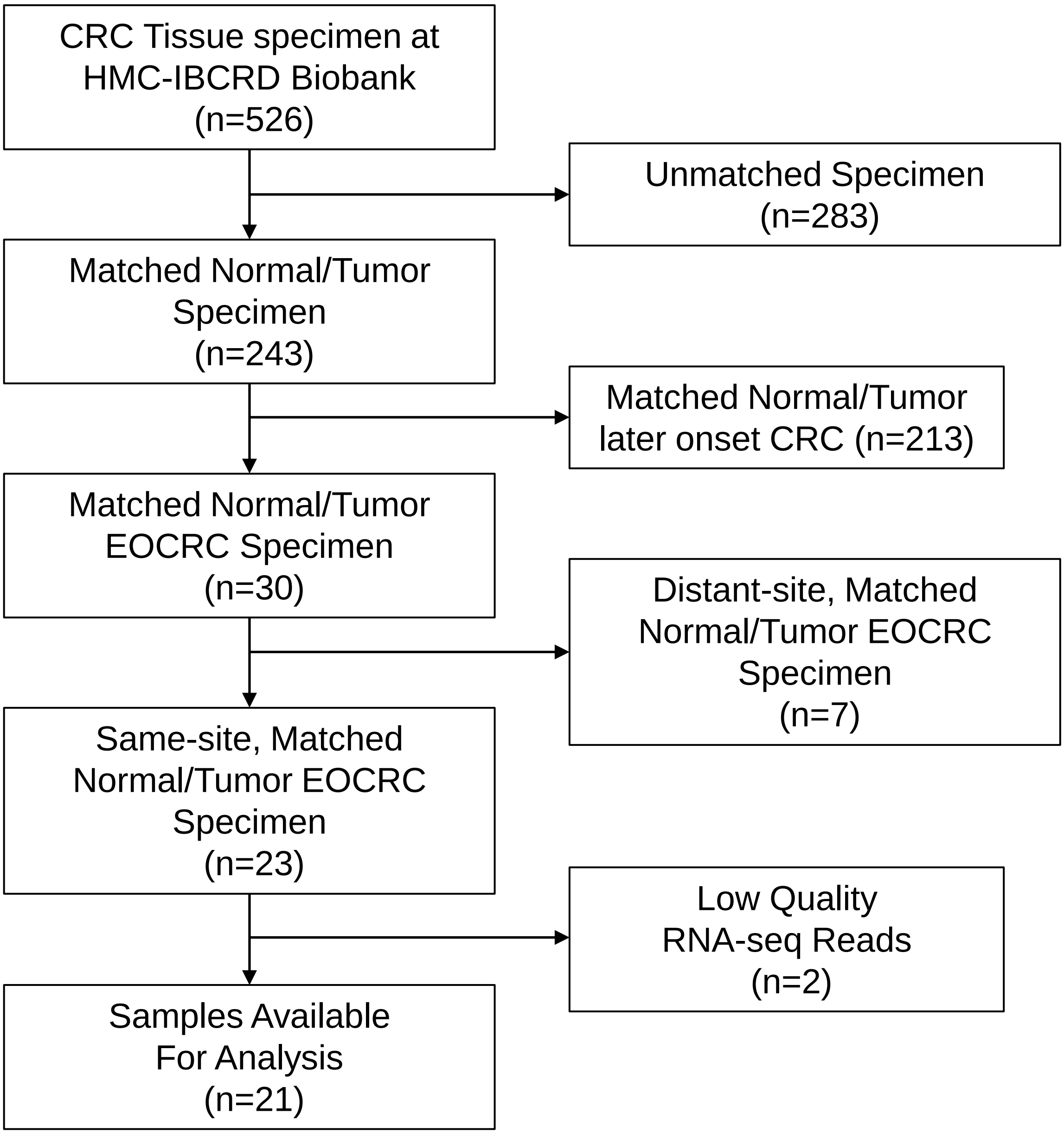

Figure S1: Flowchart of patient sample selection. Colorectal cancer (CRC) patients were selected from the Inflammatory Bowel and Colorectal Diseases Biobank (IBCRD). Early onset colorectal cancer (EOCRC) patients were under 50 years old at diagnosis, while later onset were over 50 years old at diagnosis. Tumors with matching adjacent normal colonic segments were sequenced (n=23) and FASTQC analysis showed low quality/ rRNA contamination in 2 samples, leaving 21 matched tumor/normal pairs for further analysis.

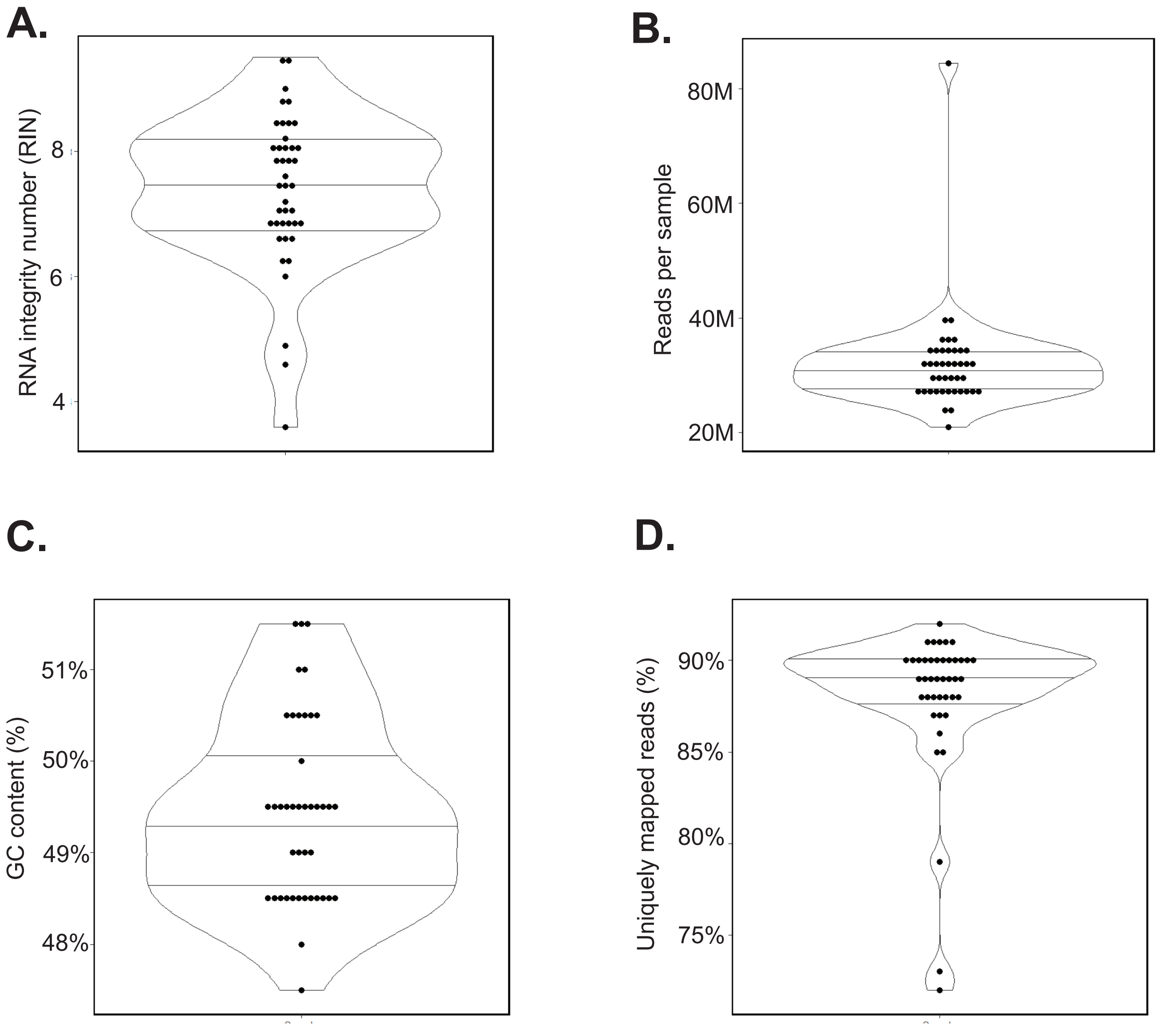

Figure S2. RNA-sequencing quality analysis of the 42 samples in this study. (**A**) RNA integrity values. (**B**) Number of reads mapped. (**C**) Percent GC content per sample. (**D**) Percent of reads mapped uniquely to the genome.

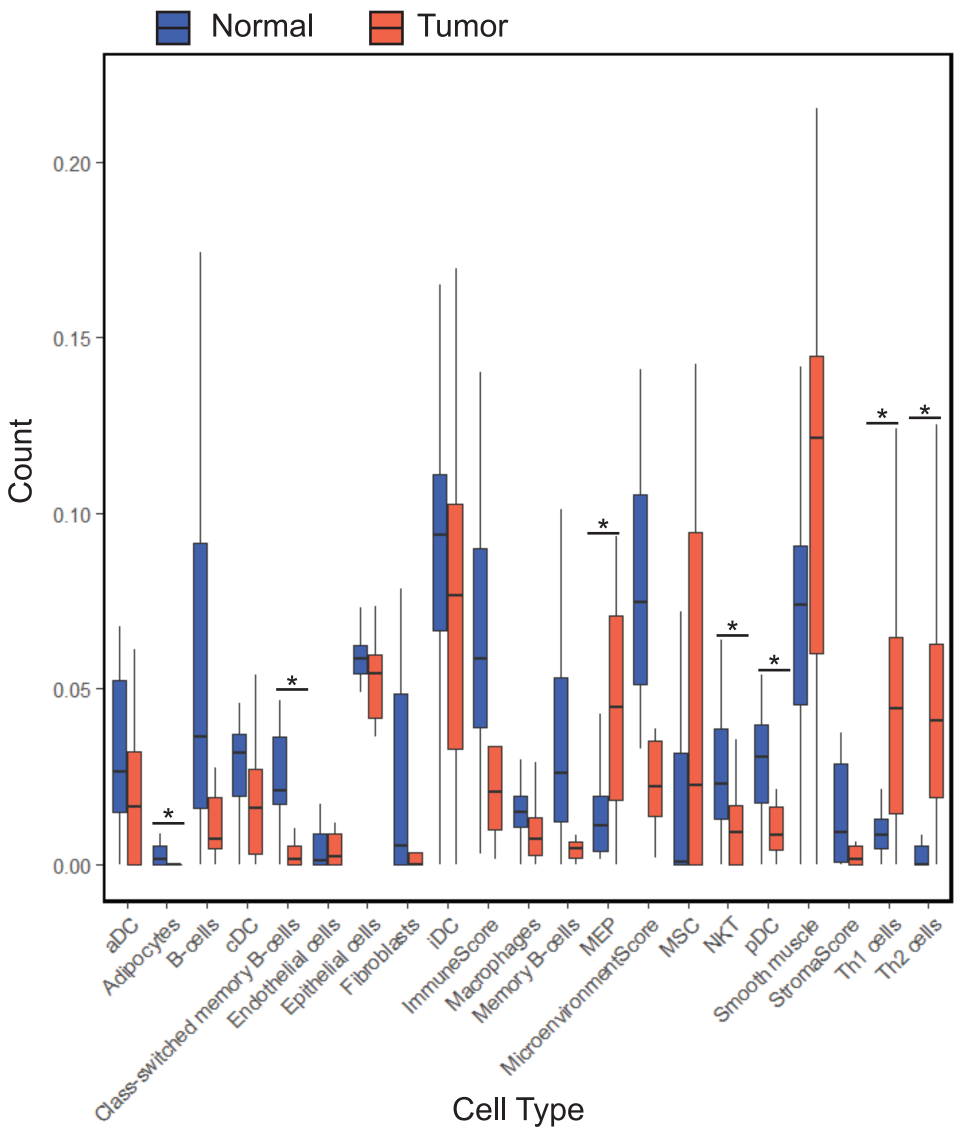

Figure S3. Cell type deconvolution (xCell) predicts a mixture of cell types in our samples. Criteria for inclusion in the graph is as follows: cells with mean counts greater than twice the mean counts of all cell types analyzed and cell types or cells with over the mean expression of all cell types and a significant (paired wilxocon test, $P < 0.01$) difference between tumor (red) and normal (blue) samples. Significant changes between tumor and normal samples ($P < 0.01$) denoted by astrisk (*).

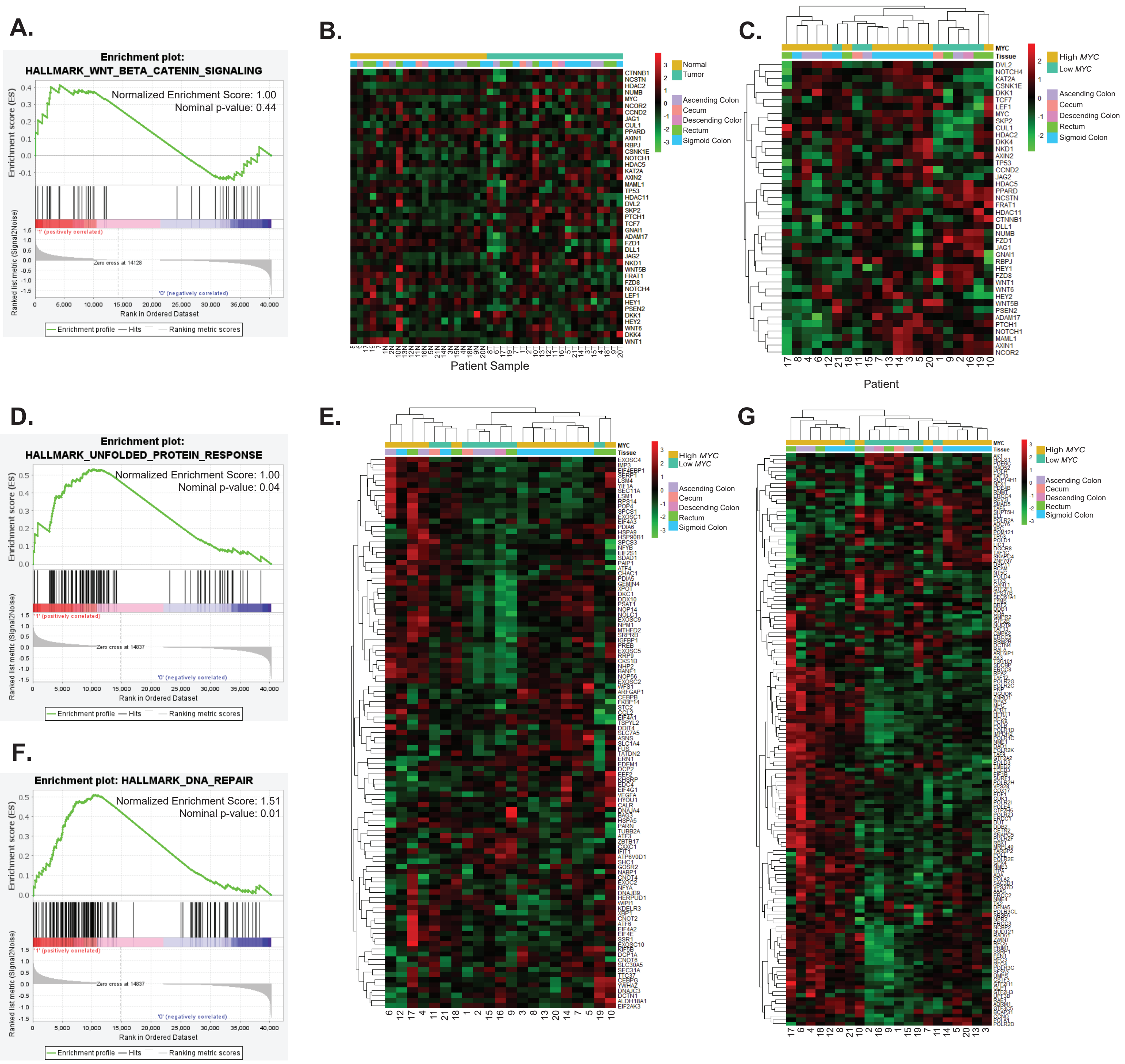

Figure S4. Hallmarks of cancer enriched in EOCRC tumors compared with controls. (**A**) GSEA of genes comprising the hallmark Wnt/beta-catenin target set in tumors compared with patient-matched control segments (nominal *P*-value = 0.44) (**B**) Heatmap of row-scaled gene expression of Wnt/beta-catenin pathway and target genes in tumors versus control segments. Patients are arbitrarily assigned 1-21 and N represents uninvolved tissues and T represents tumors. (**C**) Hierarchical clustering of differentially-expressed Wnt/beta-catenin pathway and target genes in patient-matched tumors and adjacent control segments scaled by row. (**D**) GSEA of genes comprising hallmarks of Unfolded Protein Response (UPR) in tumors compared with controls. (**E**) Heatmap of differential expression of UPR hallmarks. (**F**) GSEA of genes comprising hallmarks of DNA damage repair in patient-matched tumors and controls. (**G**) Heatmap of differential expression of DNA damage repair hallmarks in tumors compared with controls.

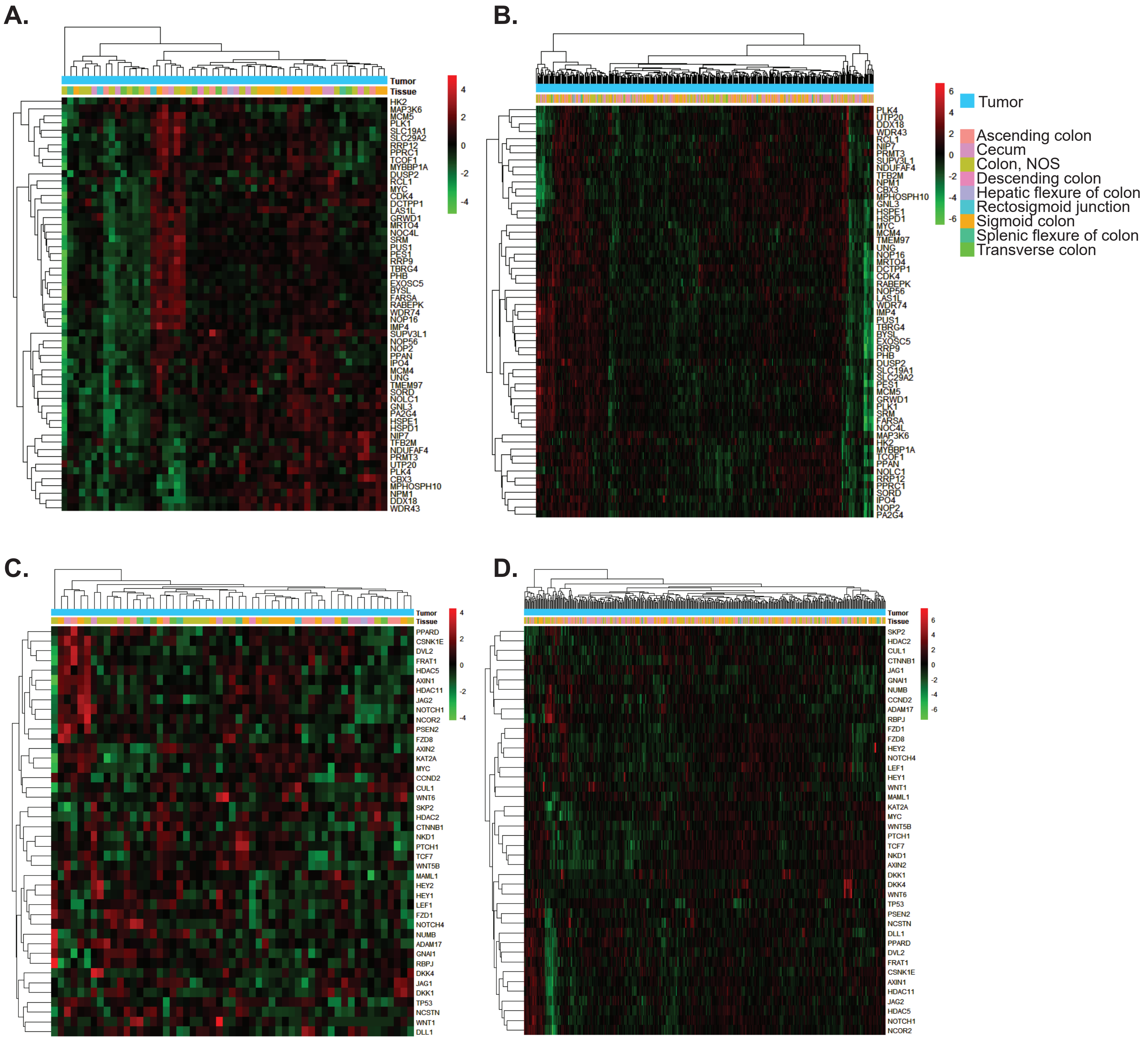

Figure S5. GSEA Hallmarks in The Cancer Genome Atlas (TGCA) colorectal adenocarcinoma dataset (COAD). Heatmaps of row-scaled gene expression of *MYC* and a subset of its downstream targets in patients diagnosed with CRC at under 18250 days (approximately 50 years old) (**A**) and patients over 18250 days old (**B**). Heatmaps of row-scaled gene expression of WNT Hallmarks of cancer in CRC tumors diagnosed under the age of 18250 days (**C**) or over 18250 days (**D**).

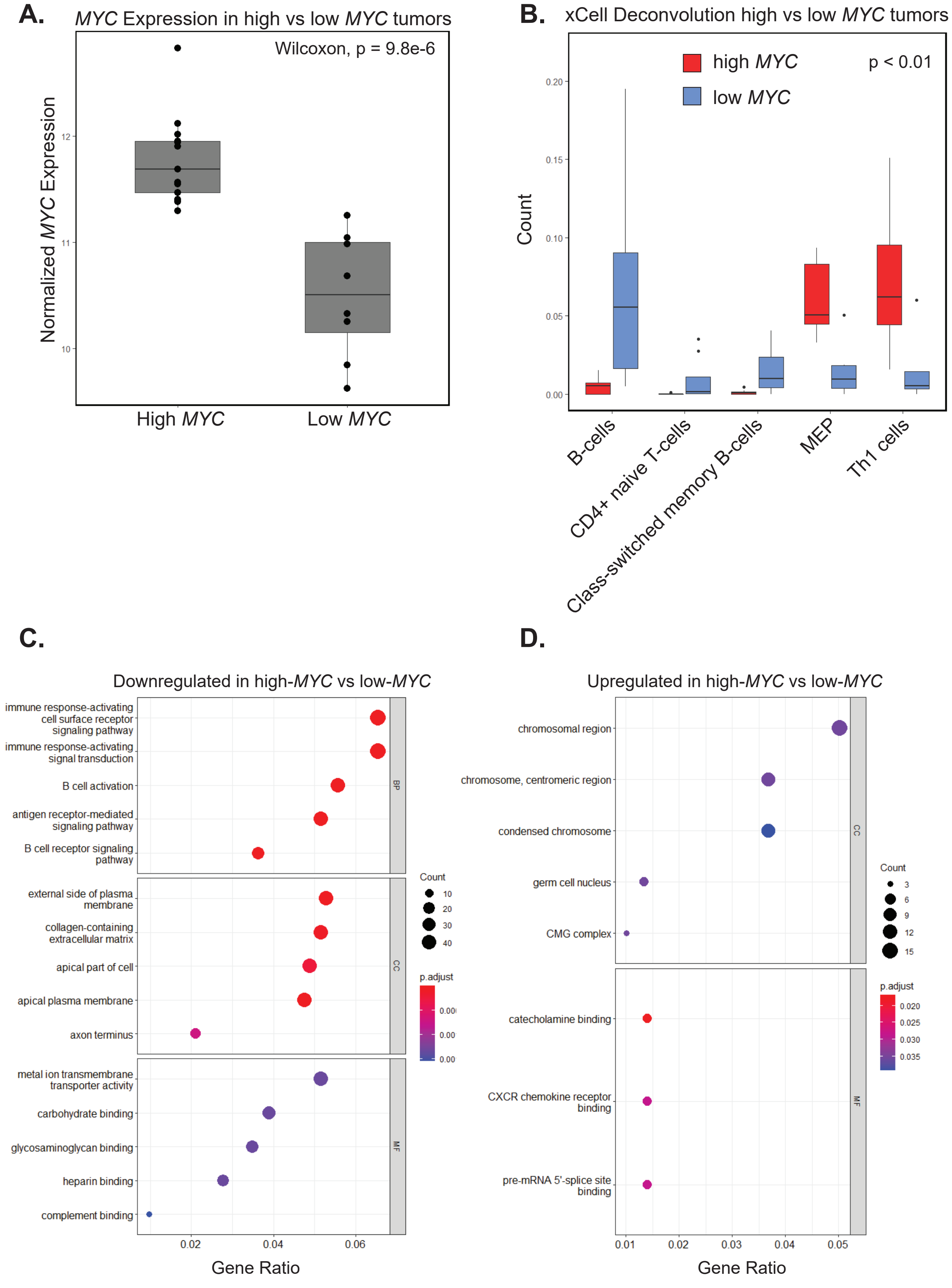

Figure S6. Comparisons of high and low *MYC* tumors. (**A**) Boxplot of rlog normalized expression of *MYC* in high and low *MYC* tumors. (**B**) Cell type deconvolution predicts significant (paired wilcoxon test, $P < 0.01$) differences between high and low *MYC* tumors. (**C**) and (**D**) show gene ontology (GO) analysis for biological process (BP), cellular component (CC), and molecular functions (MF) for singificantly downregulated (log2 fold change < -1.5) (**C**) and upregulated (log2 fold change > 1.5) (**D**) genes in high *MYC* tumors compared to low *MYC* tumors (Adjusted $P < 0.05$).

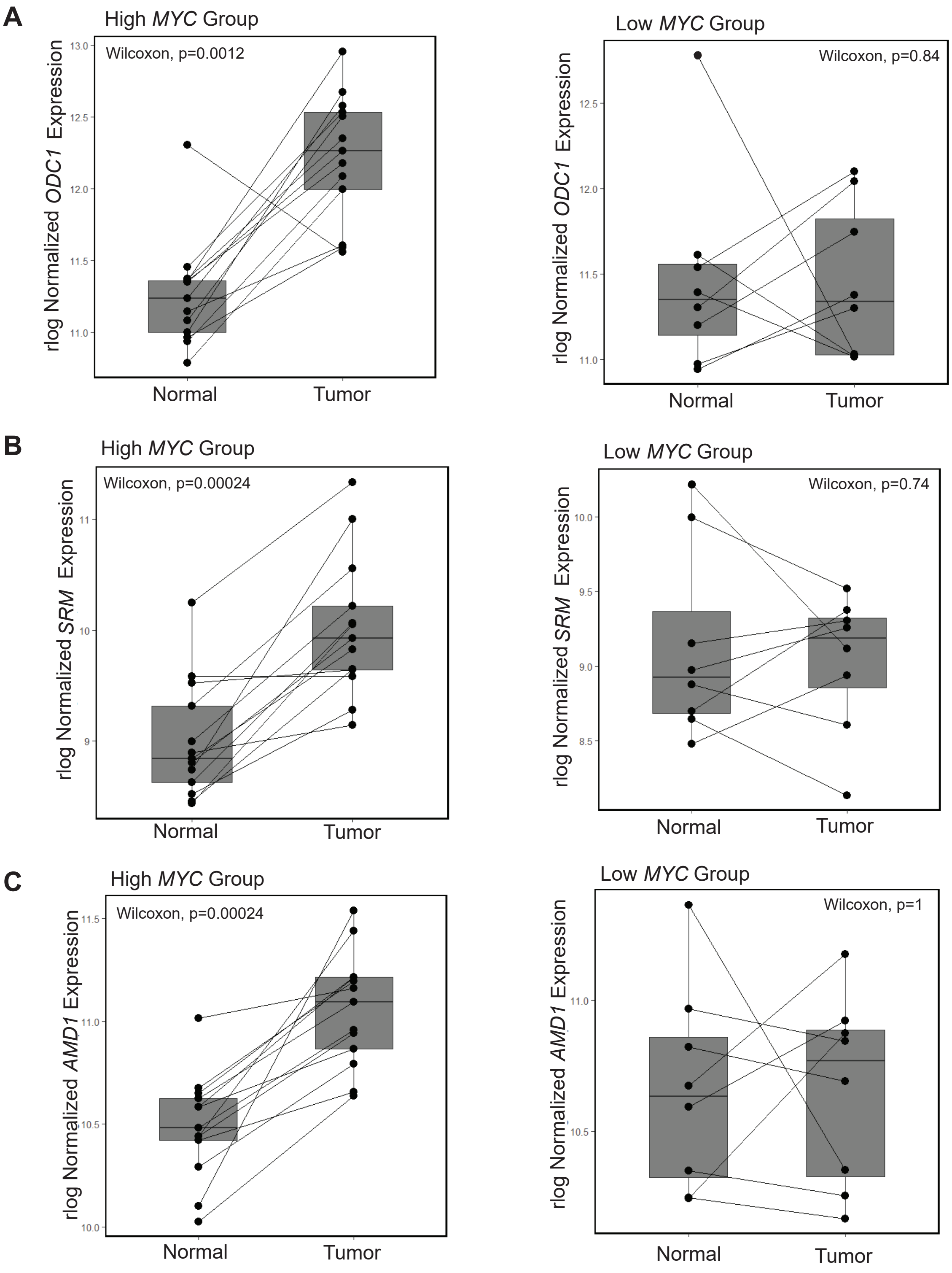

Figure S7. MYC target genes are up-regulated in high-*MYC* tumors. (**A-C**) Difference plots of rlog normalized gene expression of each gene indicated in normal samples vs. tumors in high- (left) and low-*MYC* patients. Lines connect normal and tumors from each patient and paired Wilcoxon test was performed to assess sigificant differences.

Supplementary Table S1: Upregulated gene ontology sets enriched in EOCRC tumors

GO ID	ONTOLOGY	Description	Gene Ratio	p.adjust	qvalue
**GO:0048285**	BP	organelle fission	0.069	5.74E-08	4.88E-08
**GO:0000280**	BP	nuclear division	0.068	3.73E-09	3.17E-09
**GO:0008544**	BP	epidermis development	0.062	1.61E-06	1.37E-06
**GO:0140014**	BP	mitotic nuclear division	0.050	1.75E-07	1.49E-07
**GO:0019730**	BP	antimicrobial humoral response	0.037	2.00E-09	1.70E-09
**GO:0061844**	BP	antimicrobial humoral immune response mediated by antimicrobial peptide	0.028	2.00E-09	1.70E-09
**GO:0098687**	CC	chromosomal region	0.046	7.61E-06	7.01E-06
**GO:0000775**	CC	chromosome, centromeric region	0.035	7.02E-07	6.47E-07
**GO:0000776**	CC	kinetochore	0.030	2.93E-07	2.70E-07
**GO:0000793**	CC	condensed chromosome	0.030	0.000472	0.000435
**GO:0000777**	CC	condensed chromosome kinetochore	0.025	7.02E-07	6.47E-07
**GO:0000779**	CC	condensed chromosome, centromeric region	0.025	2.33E-06	2.15E-06
**GO:0048018**	MF	receptor ligand activity	0.081	8.93E-13	8.32E-13
**GO:0030546**	MF	signaling receptor activator activity	0.081	8.93E-13	8.32E-13
**GO:0005125**	MF	cytokine activity	0.047	2.03E-09	1.89E-09
**GO:0001664**	MF	G protein-coupled receptor binding	0.044	4.65E-06	4.34E-06
**GO:0008009**	MF	chemokine activity	0.018	1.76E-06	1.64E-06
**GO:0045236**	MF	CXCR chemokine receptor binding	0.013	1.30E-07	1.21E-07

Supplementary table S1: Gene ontology (GO) sets of upregulated genes enriched in EOCRC tumors compared with adjacent normal samples. Top six results from each category are shown: biological process (BP), cellular component (CC), and molecular function (MF).

Supplementary Table S2: Gene sets enriched in EOCRC tumors

Gene Set	Normalized Enrichment Score	Nominal p-value
HALLMARK_DNA_REPAIR	1.52	0.012
HALLMARK_MYC_TARGETS_V2	1.52	0.012
HALLMARK_MYC_TARGETS_V1	1.50	0.019
HALLMARK_UNFOLDED_PROTEIN_RESPONSE	1.49	0.044
HALLMARK_ADIPOGENESIS	-1.51	0.030

Supplementary Table S2: GSEA for tumors compared with adjacent normal samples. All results with significant (nominal $P < 0.05$) shown.

Supplementary Table S3: Patient demographics associated with *MYC* copy number variation

Parameter	CNV unchanged	CNV >2	p-value
Patients, n	11	6	
Age at Diagnosis, median (IQR)	43 (39-47)	49 (47-50)	**0.007**
Sex			0.64
Male, n (%)	7 (64%)	3 (50%)	
Female, n (%)	4 (36%)	3 (50%)	
Race			1.00
White, n (%)	9 (82%)	6 (100%)	
Black, n (%)	1 (9%)	0 (0%)	
Asian, n (%)	1 (9%)	0 (0%)	
BMI			1.00
Normal or underweight, n (%)	2 (18%)	1 (17%)	
Overweight or Obese, n (%)	9 (82%)	5 (83%)	
History of Smoking[a], n (%)	6 (55%)	1 (17%)	0.30
Family History[b], n (%)	6 (55%)	3 (50%)	1.00
Located at Sigmoid or Rectum, n (%)	6 (55%)	6 (100%)	0.10
Overall Stage			0.43
I, n (%)	2 (18%)	3 (50%)	
II, n (%)	3 (27%)	0 (0%)	
III, n (%)	5 (45%)	2 (33%)	
IV, n (%)	1 (9%)	1 (17%)	
Signet/Mucinous component	2 (18%)	0 (0%)	0.51
Lymphovascular Invasion	1 (9%)	4 (67%)	**0.028**
Peri-neural Invasion	5 (45%)	1 (17%)	0.60

.

[a] Positive smoking status includes both current and former smokers at the time of surgery

[b] Positive family history for colon or rectal cancer in first degree family member
